# Tuftsin prevents the negative immunoregulation of neuropilin-1^high^CD4^+^CD25^+^Regulatory T cells and improves survival rate in septic mice

**DOI:** 10.18632/oncotarget.13235

**Published:** 2016-11-09

**Authors:** Yu-Lei Gao, Mu-Ming Yu, Song-Tao Shou, Ying Yao, Yan-Cun Liu, Li-Jun Wang, Bin Lu, Yan-Fen Chai

**Affiliations:** ^1^ Department of Emergency Medicine, Tianjin Medical University General Hospital, Tianjin 300052, P.R. China

**Keywords:** sepsis, regulatory T cells, negative immunoregulation, tuftsin, neuropilin-1

## Abstract

Our previous research showed that neuropilin (Nrp) -1^high^CD4^+^CD25^+^Regulatory T cells (Tregs) exhibited primary negative immunoregulation in sepsis induced immune dysfunction. Tuftsin is the typical ligand of Nrp-1. Herein, we investigated the potential therapeutic value and mechanisms of tuftsin in sepsis. Sepsis *per se* markedly decreased the serum concentration of tuftsin, administration of tuftsin improved the survival rate of septic mice with cecal ligation and puncture (CLP). In *vitro* study, tuftsin prevented the negative immunoregulation of Nrp-1^high^CD4^+^CD25^+^Tregs, including weakening the expression of forkhead/winged helix transcription factor (Foxp)- 3/cytotoxic T lymphocyte associated antigen (CTLA)-4, inhibiting the secretion of transforming growth factor (TGF)-β, and weakening the immunosuppressive function of Nrp-1^high^CD4^+^CD25^+^Tregs to conventional CD4^+^CD25^−^T cells. Tuftsin markedly inhibited the demethylation of Foxp3-Tregs specific demethylated region (TSDR) of Nrp-1^high^CD4^+^CD25^+^Tregs. Tuftsin could represent a new potential therapeutic agentia to improve the outcome of septic mice, and associate with preventing the negative immunoregulation of Tregs via Nrp-1.

## INTRODUCTION

Sepsis is still the leading cause of death among critically ill patients in intensive care units, and the quality of life for the survivors would usually be impaired [1–5]. As a result, there is a significant loss of immunocytes, including B/T lymphocytes, dendritic cells (DCs), gastrointestinal epithelial cells, even thymocytes at the beginning of sepsis as shown both in animal models and septic patients [6–9]. It has been noted that septic patients gradually enter into a state of immunosuppression after primary hyper-inflammatory response, and defined as immunoparalysis [2, 4, 6-7]. In recent years, investigators have become interested in the study of the mechanisms regarding immunosuppression and development of new measures to improve immunosuppression during sepsis, including activation of Tregs and apoptotic depletion of immunocytes [10].

Tregs, as a class of CD4^+^T cell subsets, are a group of specialized immune cells that play an important role in immune homeostasis [11]. With the development of sepsis, Tregs subdue inflammation and tissue damage, while they could also cause immune dysfunction, such as induction of T-lymphocytic apoptosis, inhibition of CD4^+^/CD8^+^ T--lymphocytic function, and mediation of shifting from the helper T cell (Th) 1 to Th 2 response, especially immunoparalysis via expression of CTLA-4 and membrane associated transforming growth factor-β (TGF-β^m+^), as well as anti-inflammatory cytokines (IL-10 and TGF-β) [12–17].

Recently, Nrp-1, characterized as a single-pass transmembrane glycoprotein, is an essential component of the immunological response in humans and animals, and identified as a potent surface marker for CD4^+^CD25^+^Tregs [18–21]. In addition, the expression of Nrp-1 on Tregs was correlated with the expression of Foxp-3 and suppressive capacity [22]. Our previous research demonstrated that Nrp-1^high^CD4^+^CD25^+^Tregs showed strong resilience to apoptosis and secretive ability, as well as the strongest immunosuppressive ability on CD4^+^CD25^−^ T cells. In the presence of lipopolysaccharide (LPS), the recombinant Nrp-1 polyclonal antibody reduced the demethylation of Foxp-3-TSDR. Nrp-1^high^Tregs might reveal primary negative immunoregulation in sepsis, Nrp-1 could represent a new potential therapeutic target for the study of immune regulation in sepsis. [23].

In 1970s, investigators found a natural immune modulating tetrapeptide (threonine-lysine-proline-arginine) derived from the proteolytic degradation 289-292 amino acid residues of IgG in spleen, and it was described as a phagocytosis-stimulating factor in terms of tuftsin which is the typical ligand of Nrp-1 [24–25]. The primary effect of tuftsin or tuftsin-like peptides was to enhance phagocyte respiratory burst, migration/chemotaxis ability, antigen presentation, and monocytic origin, including macrophages, neutrophils, microglia and Kupffer cells, thereby increasing antimicrobial and antitumor activities [26–30]. Recently, we demonstrated that tuftsin-derived T-peptide had potential effect on adaptive immunity in sepsis, such as lowering the suppressive ability of CD4^+^CD25^+^ Tregs on CD4^+^CD25^−^ T cells [31]. Nevertheless, the impact of sepsis on the serum concentration of tuftsin and the expression of Nrp-1 on CD4^+^CD25^+^Tregs, as well as the potential therapeutic value and mechanisms of tuftsin in sepsis remains to be elucidated.

In the present study, using the classical septic model, i.e., CLP, we demonstrated that the serum concentration of tuftsin was significantly decreased, and the expression of Nrp-1 was significantly enhanced in a grade- and time- dependent manner. Administration of tuftsin improved the survival rate of septic mice in a dose- dependent manner, especially treatment with 2 mg/kg tuftsin after CLP. In *vitro* study, tuftsin prevented the negative immunoregulation of Tregs, the primary subtype is Nrp-1^high^CD4^+^CD25^+^Tregs, including down-regulating the expression of Foxp-3/CTLA-4, inhibiting the secretion of TGF-β, and down-regulating the immunosuppressive function of Nrp-1^high^CD4^+^CD25^+^Tregs to conventional CD4^+^CD25^−^T cells. In *vitro* and *vivo* study, tuftsin markedly inhibited the demethylation of Foxp3-TSDR of Nrp-1^high^CD4^+^CD25^+^Tregs in a dose-dependent manner. Tuftsin could represent a new potential therapeutic agentia to improve the outcome of septic mice, and prevent the negative immunoregulation of regulatory T cells via Nrp-1.

## RESULT

### Sepsis markedly decreased the serum concentration of tuftsin in a grade- and time- dependent pattern

**Figure 1:**
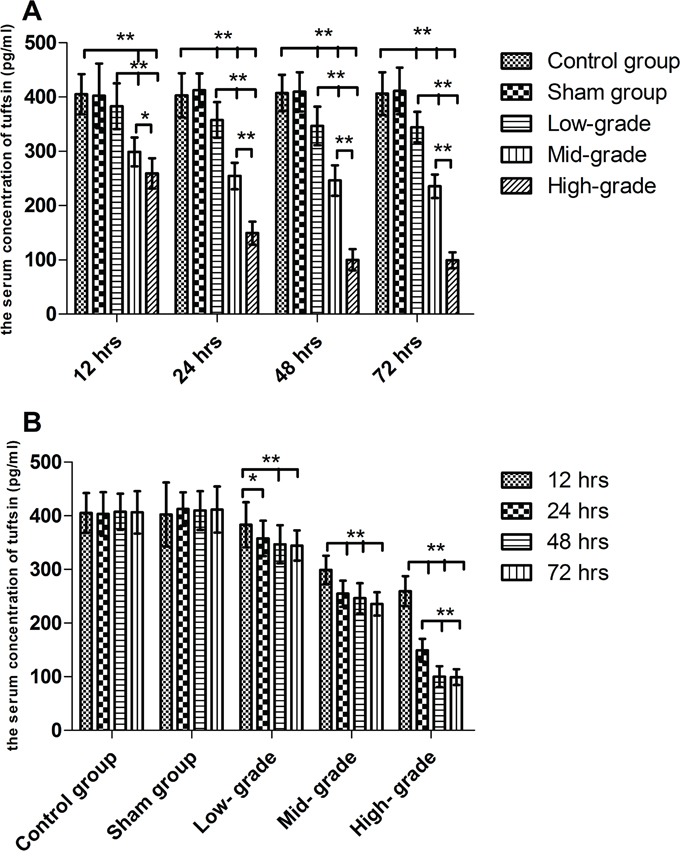
The grade- and time- dependent responses between sepsis and the serum concentration of tuftsin The results showed the effects of various severity of sepsis on the serum concentration of tuftsin after CLP **A**. The time-dependent response between various severity of sepsis and the serum concentration of tuftsin **B**. Data were represented as mean ± standard deviation (SD), and analyzed by software of SPSS 17.0 with a one-way ANOVA, n=4 per group, *P<0.05, **P<0.01.

As shown in Figure 1A, from 12 to 72 hrs, it was found that compared with the control and sham groups, the serum concentration of tuftsin was significantly decreased by sepsis (*P*<0.01). Compared with low-grade septic groups, the serum concentration of tuftsin was further decreased in mid- and high- grade septic groups (*P*<0.05 or 0.01), especially high- grade septic groups in comparison to mid-grade groups (*P*<0.01), in a grade- dependent pattern. As shown in Figure 1B, there were no differences among control and sham groups from 12 to 72 hrs (*P*>0.05). In low- and mid- grade group, the serum concentration of tuftsin was further decreased from 24 to 72 hrs in comparison to 12 hrs (*P*<0.05 or 0.01), but there were no differences from 24 to 72 hrs (*P*>0.05). In high- grade group, compared with 12 hrs, the serum concentration of tuftsin was further decreased from 24 to 72 hrs(*P*< 0.01), especially from 48 to 72 hrs in comparison to 24 hrs (*P*< 0.01).

### Sepsis markedly enhanced the expression of Nrp-1 on CD4^+^CD25^+^Tregs in a grade- and time- dependent pattern

**Figure 2:**
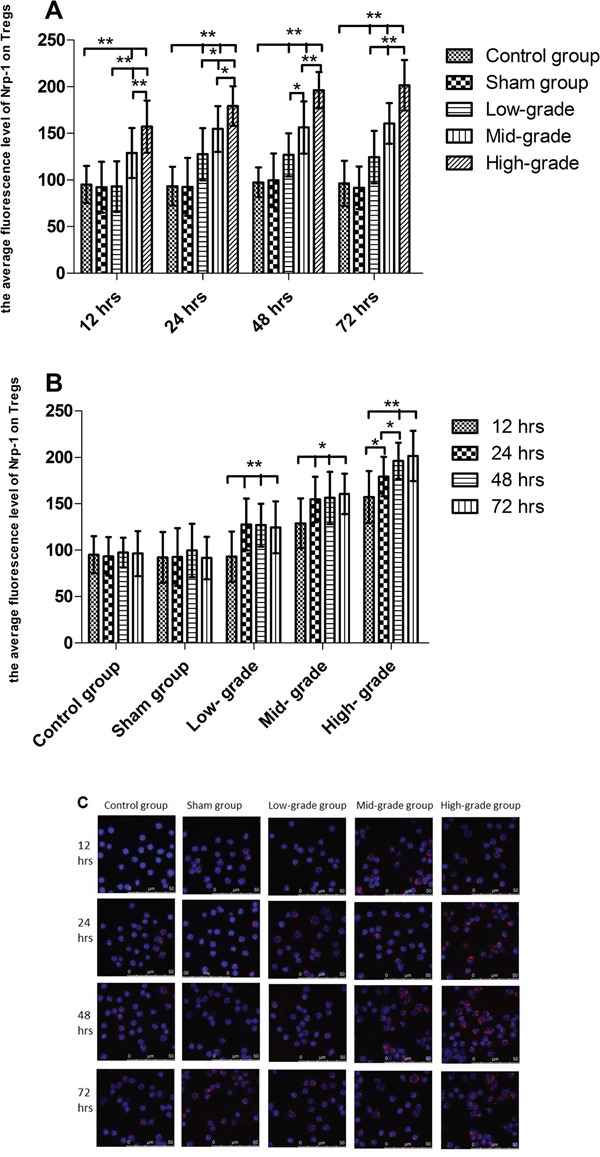
The grade- and time- dependent responses between sepsis and the expression of Nrp-1 on splenic CD4^+^CD25^+^Tregs The results showed the effects of various severity of sepsis on the average fluorescence level of Nrp-1 on splenic CD4^+^CD25^+^Tregs after CLP **A**. The time-dependent response between various severity of sepsis and the average fluorescence level of Nrp-1 **B**. Immunofluorescence confocal microscopy using antibodies to Nrp-1 **(C, red)**. Data were represented as mean ± standard deviation (SD), and analyzed by software of SPSS 17.0 with a one-way ANOVA, n=4 per group, *P<0.05, **P<0.01.

As shown in Figure 2A, from 12 to 72 hrs, compared with the control and sham groups, the expression of Nrp-1 on CD4^+^CD25^+^Tregs was significantly enhanced by sepsis (*P*<0.01). Compared with low-grade septic groups, the expression of Nrp-1 was further enhanced in mid- and high- grade septic groups (*P*<0.05 or 0.01), especially high- grade septic groups in comparison to mid-grade groups (*P*<0.05 or 0.01), in a grade- dependent pattern. As shown in Figure 2B, there were no differences between control and sham groups from 12 to 72 hrs (*P*>0.05). In low- and mid- grade group, the expression of Nrp-1 was further enhanced from 24 to 72 hrs in comparison to 12 hrs(*P*<0.05 or 0.01), but there were no differences from 24 to 72 hrs (*P*>0.05). In high- grade group, compared with 12 hrs, the expression of Nrp-1 was further enhanced from 24 to 72 hrs (*P*< 0.01), especially from 48 to 72 hrs in comparison to 24 hrs (*P*< 0.05).

### Tuftsin significantly improved the survival rate of septic mice in a dose- dependent manner

**Figure 3:**
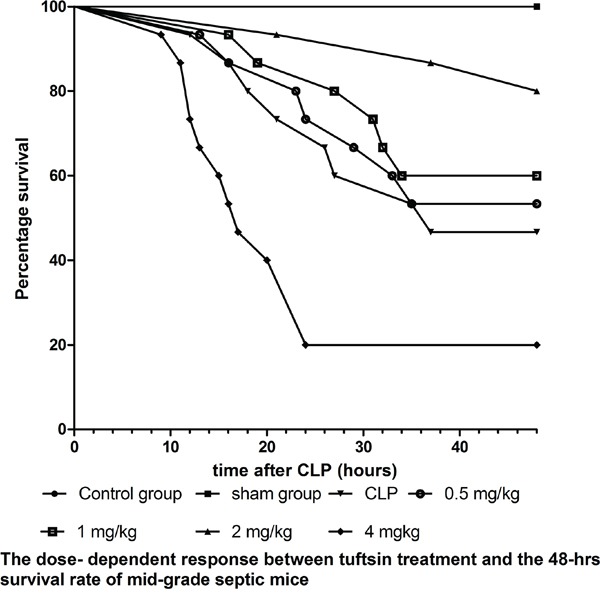
The dose-dependent response between tuftsin and the 48- hrs survival rate The survival rate was analyzed by Kaplan-Meier via the log-rank test, n=30 per group, *P*<0.05 or *P*<0.01.

We employed mid-grade septic mice to observe the dose- dependent response between tuftsin and the survival rate of septic mice. As shown in Figure 3, the 48 hrs- survival rate of CLP and administration of 0.5 mg/kg tuftsin groups were 46.67% and 53.33%, respectively, and there was no difference between them according to Kaplan-Meier analysis (*P*>0.05). Compared with CLP group, administration of 1 and 2 mg/kg tuftsin significantly increased the 48 hrs- survival rate (*P*<0.05 or 0.01), the 48 hrs- survival rate of them were 60.00% and 80.00%, especially 2mg/kg in comparison to 1 mg/kg according to Kaplan-Meier analysis (*P*<0.01). However, the 48 hrs- survival rate of 4 mg/kg tuftsin group (20.00%) was obviously decreased (*P*<0.01). The 24 hrs- survival rate of CLP and administration of various doses tuftsin groups were 73.33%, 73.33%, 86.67%, 93.33% and 20%, administration of 2 mg/kg tuftsin significantly increased the 24 hrs- survival rate in comparison to other groups (*P*<0.05 or 0.01). Therefore, treatment with 2 mg/kg tuftsin after CLP was the most appropriate dose.

### Tuftsin weakened the expression of Foxp-3 of Nrp-1^high^CD4^+^CD25^+^Tregs

**Figure 4:**
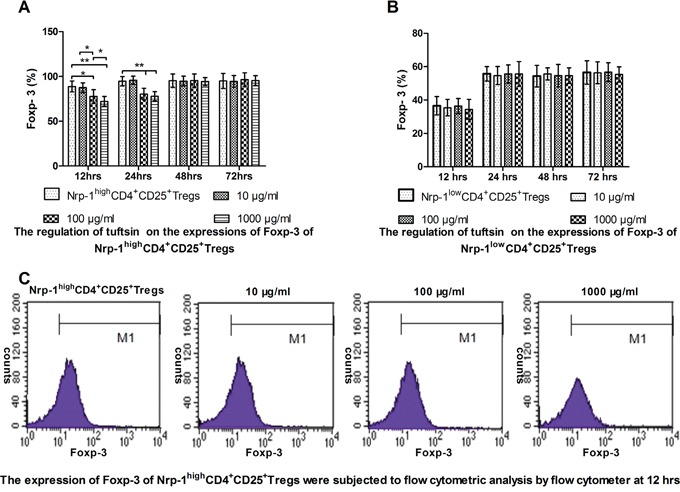
The impact of tuftsin on the expressions of Foxp-3 Tuftsin weakened the expression of Foxp-3 of Nrp-1^high^CD4^+^CD25^+^Tregs from 12 to 24 hrs **A**. Tuftsin had no effect on the expression of Foxp-3 of Nrp-1^low^CD4^+^CD25^+^Tregs **B**. The expression of Foxp-3 of Nrp-1^high^CD4^+^CD25^+^Tregs were subjected to flow cytometric analysis by flow cytometer at 12 hrs **C**. Data were represented as mean ± standard deviation (SD), and analyzed by software of SPSS 17.0 with a one-way ANOVA, n=4 per group, *P<0.05, **P<0.01.

In *vitro* study, CD4^+^CD25^+^Tregs were selected for Nrp-1^high^CD4^+^CD25^+^Tregs and Nrp-1^low^CD4^+^CD25^+^Tregs. As shown in Figure 4A, when treated with various doses of tuftsin for 12, 24, 48 and 72 hrs, the expressions of Foxp-3 on Nrp-1^high^CD4^+^CD25^+^Tregs was significantly weakened from 12 hrs to 24 hrs in comparison to control group (*P*<0.05 or 0.01), especially the dose of 1000 μg/ml at 12 hrs in comparison to 100 μg/ml (*P*<0.05), and there were no difference from 48 hrs to 72 hrs (*P*>0.05). As shown in Figure 4B, when Nrp-1^low^CD4^+^CD25^+^Tregs were treated with various doses of tuftsin, there were no difference on the expressions of Foxp-3 among all groups from 12 hrs to 72 hrs (*P*>0.05).

### Tuftsin weakened the expression of CTLA-4 of Nrp-1^high^CD4^+^CD25^+^Tregs

**Figure 5:**
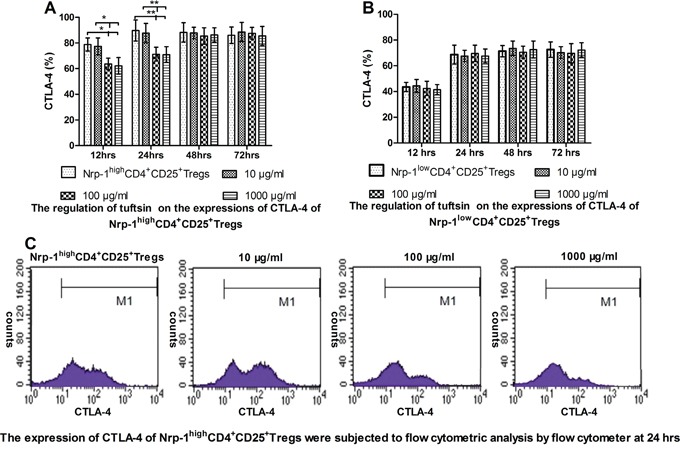
The impact of tuftsin on the expressions of CTLA-4 Tuftsin weakened the expression of CTLA-4 of Nrp-1^high^CD4^+^CD25^+^Tregs from 12 to 24 hrs **A**. Tuftsin had no effect on the expression of CTLA-4 of Nrp-1^low^CD4^+^CD25^+^Tregs **B**. The expression of CTLA-4 of Nrp-1^high^CD4^+^CD25^+^Tregs were subjected to flow cytometric analysis by flow cytometer at 24 hrs **C**. Data were represented as mean ± standard deviation (SD), and analyzed by software of SPSS 17.0 with a one-way ANOVA, n=4 per group, *P<0.05, **P<0.01.

In *vitro* study, As shown in Figure 5A, when treated with various doses of tuftsin for 12, 24, 48 and 72 hrs, the expressions of CTLA-4 on Nrp-1^high^CD4^+^CD25^+^Tregs was significantly weakened from 12 hrs to 24 hrs in the doses of 100 and 1000 μg/ml in comparison to control and 10 μg/ml groups (*P*<0.05 or 0.01), and there were no difference between the doses of 100 and 1000 μg/ml (*P*>0.05). As shown in Figure 5B, when Nrp-1^low^CD4^+^CD25^+^Tregs were treated with various doses of tuftsin, there were no difference on the expressions of CTLA-4 among all groups from 12 hrs to 72 hrs (*P*>0.05).

### Tuftsin inhibited the secretion of TGF-β from Nrp-1^high^CD4^+^CD25^+^Tregs

**Figure 6:**
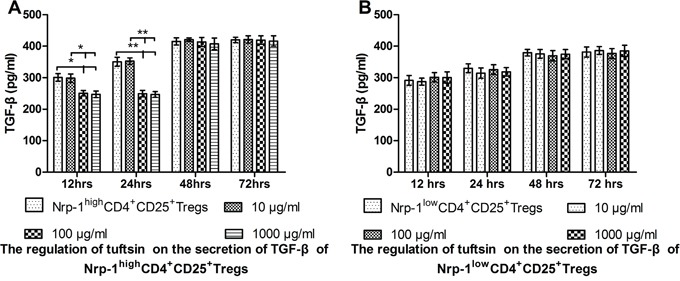
The impact of tuftsin on the secretion of TGF-β Tuftsin inhibited the secretion of TGF-β from Nrp-1^high^CD4^+^CD25^+^Tregs from 12 to 24 hrs **A**. Tuftsin had no effect on the secretion of TGF-β from Nrp-1^low^CD4^+^CD25^+^Tregs **B**. Data were represented as mean ± standard deviation (SD), and analyzed by software of SPSS 17.0 with a one-way ANOVA, n=4 per group, *P<0.05, **P<0.01.

In *vitro* study, As shown in Figure 6A, when treated with various doses of tuftsin for 12, 24, 48 and 72 hrs, the secretion of TGF-β from Nrp-1^high^CD4^+^CD25^+^Tregs was significantly inhibited from 12 hrs to 24 hrs in the doses of 100 and 1000 μg/ml in comparison to control and 10 μg/ml groups (*P*<0.05 or 0.01), and there were no difference between the doses of 100 and 1000 μg/ml (*P*>0.05). As shown in Figure 6B, when Nrp-1^low^CD4^+^CD25^+^Tregs were treated with various doses of tuftsin, there was no difference on the secretion of TGF-β among all groups from 12 hrs to 72 hrs (*P*>0.05).

### Tuftsin weakened the immunosuppressive function of Nrp-1^high^CD4^+^CD25^+^Tregs

**Figure 7:**
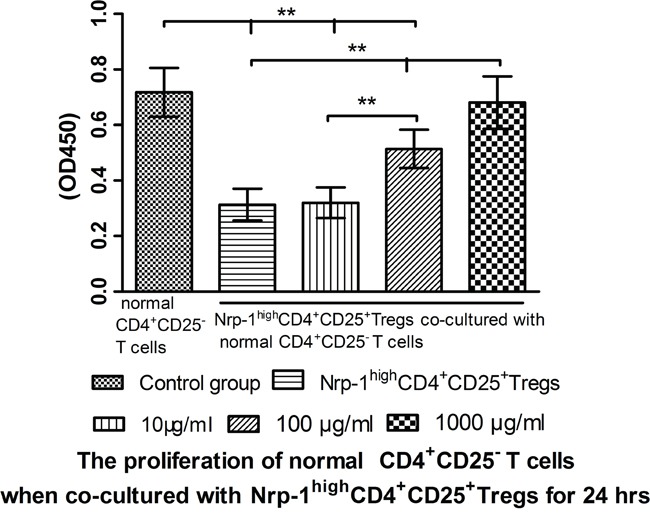
The regulating ability of tuftsin on Nrp-1^high^CD4^+^CD25^+^Tregs to the proliferative activity of conventional CD4^+^CD25^−^ T cells Tuftsin weakened the suppressive function of Nrp-1^high^CD4^+^CD25^+^Tregs to the proliferative activity of conventional CD4^+^CD25^−^ T cells. Data were represented as mean ± standard deviation (SD), and analyzed by software of SPSS 17.0 with a one-way ANOVA, n=4 per group, *P<0.05, **P<0.01.

**Figure 8:**
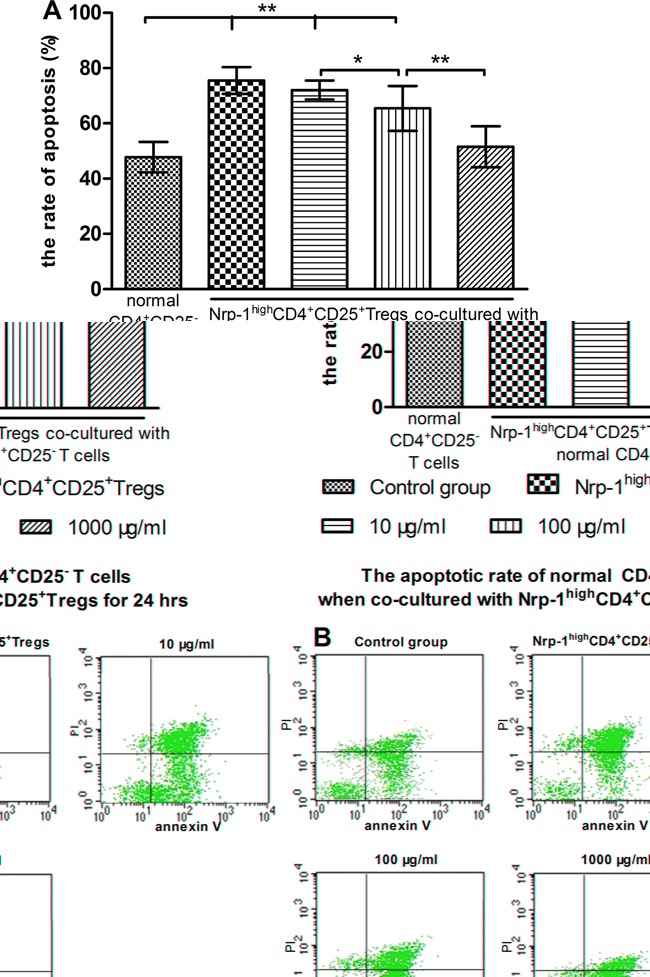
The regulating ability of tuftsin on Nrp-1^high^CD4^+^CD25^+^Tregs to the apoptosis of conventional CD4^+^CD25^−^ T cells Tuftsin weakened the suppressive function of Nrp-1^high^CD4^+^CD25^+^Tregs to the apoptosis of conventional CD4^+^CD25^−^ T cells **A**. The apoptotic rate of CD4^+^CD25^−^ T cells was analyzed with annexin-V-FITC/PI flow cytometry at 24 hours after co-cultured with Nrp-1^high^CD4^+^CD25^+^Tregs **B**. Data were represented as mean ± standard deviation (SD), and analyzed by software of SPSS 17.0 with a one-way ANOVA, n=4 per group, *P<0.05, **P<0.01.

**Figure 9:**
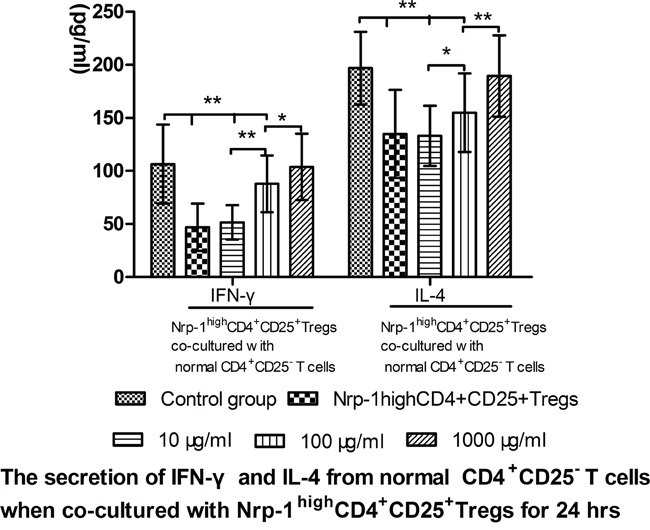
The regulating ability of tuftsin on Nrp-1^high^CD4^+^CD25^+^Tregs to the secretive ability of conventional CD4^+^CD25^−^ T cells Tuftsin weakened the suppressive function of Nrp-1^high^CD4^+^CD25^+^Tregs to the secretive ability of conventional CD4^+^CD25^−^ T cells. Data were represented as mean ± standard deviation (SD), and analyzed by software of SPSS 17.0 with a one-way ANOVA, n=4 per group, *P<0.05, **P<0.01.

In *vitro* study, we investigated the effect of tuftsin on the immunosuppressive function of Nrp-1^high^CD4^+^CD25^+^Tregs to conventional CD4^+^CD25^−^ T cells. Nrp-1^high^CD4^+^CD25^+^Tregs were stimulated for 24 hrs by various doses tuftsin in the presence of LPS, and then they were co-cultured with conventional CD4^+^CD25^−^T cells for 24 in a ratio of 1:1, respectively. As shown in figure 7-9, Nrp-1^high^CD4^+^CD25^+^Tregs without tuftsin treatment significantly inhibited the proliferative activity (Figure 7) and the secretive ability [including interferon (IFN)-γ and IL-4, figure 9] of CD4^+^CD25^−^ T cells, but increased the apoptosis of CD4^+^CD25^−^T cells (Figure 8) compared with control group (*P*<0.01). After Nrp-1^high^CD4^+^CD25^+^Tregs was treated by various doses tuftsin for 24 hours, and co-cultured with CD4^+^CD25^−^ T cells, the suppressive capacity of CD4^+^CD25^+^Tregs on CD4^+^CD25^−^ T cells was obviously diminished (*P*<0.05 or 0.01), especially 1000 μg/ml(*P*<0.01), and in a dose- dependent manner (*P*<0.05 or 0.01).

### Tuftsin inhibited the demethylation level of Foxp3-TSDR

**Figure 10:**
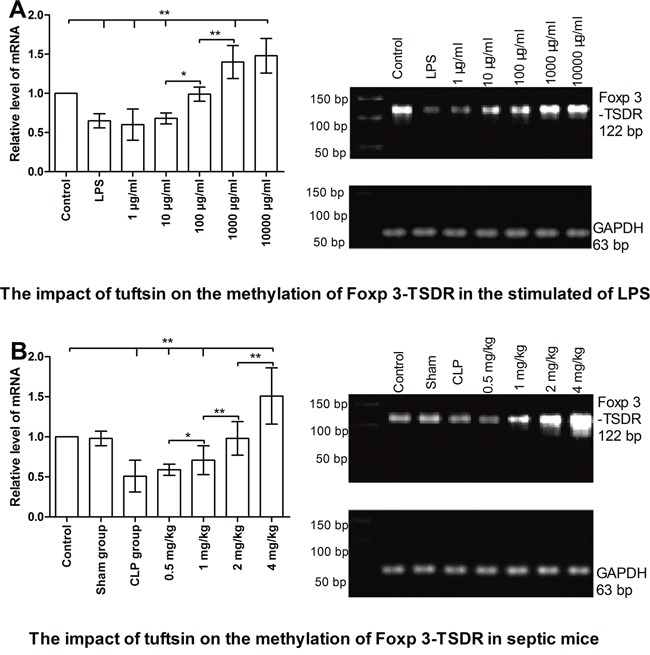
The impact of tuftsin on the methylation of Foxp 3-TSDR of Nrp-1^high^CD4^+^CD25^+^Tregs Tuftsin markedly promoted the methylation of Foxp3-TSDR in the stimulated of LPS at 24 hours in a dose-dependent manner **A**. Tuftsin markedly promoted the methylation of Foxp3-TSDR in splenic Nrp-1^high^CD4^+^CD25^+^ Tregs of septic mice at 24 hours in a dose-dependent manner **B**. Data were represented as mean ± standard deviation (SD), and analyzed by software of SPSS 17.0 with a one-way ANOVA, n=4 per group, *P<0.05, **P<0.01.

As shown in Figure 10 A, *in vitro* study, the demethylation level of Foxp3-TSDR in Nrp-1^high^CD4^+^CD25^+^ Tregs was significantly increased in the stimulated of LPS compared with control group for 24 hours using methylation-sensitive RT- PCR (*P*<0.01). Tuftsin had an obvious ability to promote the methylation level of Foxp3-TSDR, and in a dose-dependent manner, especially 1000 and 10000 μg/ml (*P*<0.05 or 0.01). As shown in Figure 10 B, *in vivo* study, the demethylation level of Foxp3-TSDR in splenic Nrp-1^high^CD4^+^CD25^+^ Tregs was significantly increased in the CLP group compared with control and sham groups for 24 hours (*P*<0.01). Tuftsin had an obvious ability to promote the methylation level of Foxp3-TSDR, and in a dose-dependent manner, especially 4 mg/kg (*P*<0.05 or 0.01). Administration of 2 mg/kg tuftsin could return the methylation level to control group (*P*>0.05).

## DISCUSSION

The broad activities of tuftsin on inherent immunocytes, especially neutrophils, microgliaes and macrophages, made the peptide as a potential therapeutic ability for immunoregulation. It binds to neutrophils and macrophages to stimulate their phagocytic activity which play a role in protecting against infections, for example killing the intracellular protozoan Leishmania major [25–28, 30]. In 1984, Baker CC and his colleague firstly suggested that tuftsin can confer protection from sepsis in animals with or without splenectomy [29]. In the current study, we reported that sepsis *per se* markedly decreased the serum concentration of tuftsin in a grade- and time- dependent pattern, administration of tuftsin improved the survival rate of septic mice after CLP, especially 2 mg/kg, which was in accordance with the findings of Baker CC.

Recent studies have shown that Nrp-1 is identified as a receptor for tuftsin on microglial cells and endothelial cells [25, 28]. Nrp-1, which was highly expressed on natural Tregs, but lowly expressed on induced Tregs and not expressed on CD4^+^CD25^−^ cells, as a good marker to distinguish natural and induced Tregs [18–21]. More interestingly, another study showed a population of Nrp-1^+^Tregs in human lymph nodes together with the positive expression of Foxp-3 that inhibited the proliferative activity of T cells [32]. In the current study, we first reported that sepsis *per se* markedly promoted the expression of Nrp-1 on CD4^+^CD25^+^Tregs in a grade- and time- dependent manner. Foxp-3, which is still the main intracellular marker for identification of Tregs, is a distinctive transcriptional factor of Tregs, and it is also critical for their function, differentiation, and maintenance [11, 13, 31]. Our previous study demonstrated that a significantly increased expression of Foxp-3 in Tregs was positively correlated to the mortality of burn-induced septic mice. The expression of Nrp-1 on Tregs was obviously correlated to the expression of Foxp-3 and the mortality of CLP-induced septic mice. Nrp-1 had the ability to preserve the negative immunoregulation of Tregs in sepsis [14–17, 23]. In the current *vitro* study, CD4^+^CD25^+^Tregs were selected for Nrp-1^high^CD4^+^CD25^+^Tregs and Nrp-1^low^CD4^+^CD25^+^Tregs, tuftsin weakened the expression of Foxp-3 of Nrp-1^high^CD4^+^CD25^+^Tregs from 12 hrs to 24 hrs, especially the dose of 1000 μg/ml at 12 hrs, which was in accordance with our previously research [31]. However, when Nrp-1^low^CD4^+^CD25^+^Tregs were treated with various doses of tuftsin, there were no difference on the expressions of Foxp-3 among all groups from 12 hrs to 72 hrs. This suggested that Nrp-1 is the primary receptor of tuftsin on Tregs in the environment of sepsis.

It has been known that immune dysfunction of CD4^+^ T lymphocytes is one of the primary cellular mechanisms in sepsis-induced immunosuppressive state [33]. Immediate observation of specimens from spleen, thymus, and lung in septic patients who died in intensive care units, or those from murine CLP model showed a profound, progressive, apoptosis-induced loss of adaptive immunocytes, thereby resulting in a decrease in ability of producing antibodies and clearing life-threatening pathogens [6-7, 34-36]. It is well known that the activated CD4^+^ T cells can mainly differentiate into Th1 and Th2, and they mainly produced IFN-γ and IL-4, respectively [34]. A shift to Th2 response was noted to be corroborated sepsis-induced immunosuppression, and the secretion of Th1 associated cytokines was thereby impaired, on the other hand, the secretion of Th2 associated cytokines was increased during sepsis, and these phenomena were obviously correlated with the outcome of septic complications [7, 36]. Our previous study suggested that Nrp-1^high^CD4^+^CD25^+^Tregs had the strongest ability to inhibit the proliferation and the cytokines secretion, but increase the apoptosis of CD4^+^CD25^−^T cells [23]. In the current *vitro* study, we showed that tuftsin has the ability to weaken the immunosuppressive function of Nrp-^1high^CD4^+^CD25^+^Tregs to conventional CD4^+^CD25^−^T cells, including increased the proliferation and the cytokines secretion , as well as decreased the apoptosis of CD4^+^CD25^−^T cells.

With the development of sepsis, Tregs can mainly inhibit the activation of T lymphocytes, especially CD4^+^T lymphocytes through various suppressive mechanisms. Accumulated evidence has shown that a combination of Foxp-3, CTLA-4, TGF-β^m+^, and inhibitory cytokines (IL-10 and TGF-β) might serve as active markers for Tregs in the process of sepsis [6-7, 13, 34-35]. Our previous study demonstrated that sepsis could obviously promote the negative immunoregulation of CD4^+^CD25^+^Tregs and Nrp-1^high^CD4^+^CD25^+^Tregs, especially Nrp-1^high^CD4^+^CD25^+^Tregs, but weakened the negative immunoregulation of Nrp-1^low^CD4^+^CD25^+^Tregs, which correlated to the expressions of Foxp-3/CTLA-4, as well as the secretion of IL-10 and TGF-β [23]. In the current *vitro* study, we showed that tuftsin could weaken the expression of Foxp-3 and CTLA-4, as well as significantly lower the secretion of TGF-β of Nrp-1^high^CD4^+^CD25^+^Tregs. These findings were in accordance with the results of our previous studies that down-regulation of activity of CD4^+^CD25^+^Tregs through *Astragalus Polysaccharides* or high mobility group box (HMGB)-1 protein marked enhanced cell-mediated immunity by modulating the proliferation of CD4^+^CD25^−^ T cells and the polarization of helper T cells, which was associated with improvement in outcome of burn-induced septic mice and decreasing the probability of secondary infection with *P. aeruginosa* [14–16].

We have reported that the percentage and stability of Tregs were higher in septic patients and mice septic models than those without sepsis. A reduction in the percentage and stability of Tregs was accompanied by an improvement in survival rate and immune dysfunction of T lymphocytes in septic mice [14–17]. It has been documented that the stability of Tregs includes the stability of Foxp-3 expression and negative immunoregulation in sepsis, which is crucially dependent on the demethylation status of the Foxp3-TSDR [13]. Our previous study suggested that recombinant Nrp-1 polyclonal antibody could decrease the demethylation of Foxp3-TSDR in the presence of LPS, thus, Nrp-1 could represent a new potential therapeutic target, at least via regulating the stability of Tregs, for the study of immune regulation in sepsis [23]. In the current *vitro* and *vivo* study, tuftsin had the ability to inhibit the demethylation level of Foxp3-TSDR, which was in accordance with our previous findings.

## MATERIALS AND METHODS

### Animals

Inbred male BALB/c mice (Laboratory Animal Center of Chinese Academy of Medical Sciences, No. SCXK-Jing-2009-0007, Beijing, China), 6-8 weeks old, 20±2 g, were used in the present study.

### Medium and reagents

The purity of tuftsin was 99.5%. Dry preparation of tuftsin was stored at -20°C, and it was dissolved to different concentrations in RPMI1640 with 10% fetal calf serum (FCS) or 0.9% sterile saline solution before use. The medium used throughout the *in vitro* experiment was RPMI1640 (containing 100 U/ml penicillin, 100 μl/ml streptomycin and 1.5 mM glutamine) with 10% heat-inactivated FCS. CD4^+^CD25^+^ regulatory T cell isolation kits [containing 1 ml cocktail of biotin-conjugated monoclonal anti-mouse CD8a, CD11b, CD45R, CD49b and Ter-119, 2 ml anti-biotin microbeads, 1 ml phycoeryanate (PE)-conjugated mouse CD25 and 1 ml anti-PE microbeads]. LD/SM columns were purchased from Miltenyi Biotec GmbH, Bergisch Gladbach, Germany. Cell counting kit-8 (CCK-8) was used to determine the proliferative activity of CD4^+^CD25^−^ T cells, and it was purchased from Dojindo, Kumamoto, Japan. Annexin V-fluorescein isothiocyanate apoptosis kit [containing 50 ml binding buffer, 500 μl annexinV-FITC and 250 μl propidium iodide (PI)] was purchased from Nanjing Keygen Biotech, Nanjing, China. LPS from *Escherichia coli* 0111:B4 was purchased from Sigma, St. Louis, MO. Purified hamster anti-mouse CD3e and CD28 were purchased from BD Pharmingen, San Diego, CA. Antibodies used for flow cytometric analysis, including FITC-conjugated anti-mouse/rat- Foxp-3, FITC- conjugated anti-mouse CD152 (CTLA-4), FITC-conjugated anti-mouse/rat-CD4 and PE-conjugated anti-mouse/ rate-CD25, were purchased from eBioscience, San Diego, CA. Enzyme-linked immunosorbent assay (ELISA) kits for tuftsin, interferon (IFN)-γ, IL-4, and TGF-β were purchased from Excell Biol, Shanghai, China. Ketamine and Su-Mianxin-II (containing 2,4-xylazole, ethylenediaminetetraacetic acid, dihydroetopine and haloperidol) were purchased from China Academy of Military Medical Sciences, Beijing, China, and they were used as anesthesia for animals.

### Isolation of splenic CD4^+^CD25^+^Tregs, CD4^+^CD25^−^Tcells, Nrp-1^high^CD4^+^CD25^+^Tregs, and Nrp-1^low^CD4^+^CD25^+^Tregs

Spleens were harvested and prepared as single cell suspension by passing through a 30 μm stainless steel mesh twice, and then treated with Ficoll-Paque density gradient centrifugation. CD4^+^CD25^+^Tregs and CD4^+^CD25^−^T cells were isolated from mononuclear cells using mouse CD4^+^CD25^+^Treg isolation kit and MiniMACS™ separator (Miltenyi Biotec GmbH, Bergisch Gladbach, Germany) according to manufacturer's instructions. CD4^+^CD25^+^Tregs were incubated with a rabbit anti-mouse Nrp-1 antibody (Abcam, Cambridge, MA) for 20 minutes at 4°C, washed and incubated with goat anti-rabbit IgG microbeads for 30 minutes at 4°C, and selected for Nrp-1^high^CD4^+^CD25^+^Tregs and Nrp-1^low^CD4^+^CD25^+^Tregs by MiniMACS™ separator according to manufacturer's instructions. Isolated cells were cultured in RPMI 1640 supplemented with 10% FCS.

### Sepsis model

After being anesthetized, a 0.5 cm incision was made on the abdomen of mice, and the cecum was exposed. The cecum at the designated position between its distal pole and ileocecal junction was ligated for the desired degree of sepsis: 1/3 for low-grade sepsis, 2/3 for mid-grade sepsis, and ligated ileocecal junction for high-grade sepsis. A single puncture was made through the cecum. The diameter of needle was 0.6 mm, which was used to induce CLP customarily in such experiment. The abdominal incision was closed using simple running sutures. Control group was only anesthetized. A sham operation ( including laparotomy and exposing the cecum without any further manipulation) was performed as sham group. All the mice were given subcutaneous injection of 0.9% sterile saline solution in 40 ml/kg body weight after CLP.

### Experimental design

150 mice were used to investigate the severity- and time- dependent response among the serum concentration of tuftsin, and the expression of Nrp-1 of Tregs, they were divided into five groups: control group, sham group, and three different CLP groups (low-grade, mid-grade, and high-grade), with 30 mice in each group. With the optimal degree of sepsis, another 210 mice were employed to observe the dose- dependent response between tuftsin (0.5, 1, 2 and 4 mg/kg) and the 48 hours (hrs)- survival rate of septic mice. The first administration of tuftsin was immediately after CLP, and tuftsin was given again at 12 hours.

*In vitro* study, Nrp-1^high^CD4^+^CD25^+^Tregs and Nrp-1^low^CD4^+^CD25^+^Tregs were subsequently seeded on 96-well (2×10^5^/well) cell culture plates, and they were treated with anti-CD3 (5 μg/ml) and anti-CD28 (2 μg/ml) antibody for polyclonal activation of T cells, respectively. Cells were then stimulated with tuftsin for different intervals of 12, 24, 48 and 72 hrs or in different doses of 1, 10, 100, 1000 and 10000 μg/ml with LPS (100 ng/ml). Nrp-1^high^CD4^+^CD25^+^Tregs were stimulated for 24 hrs by tuftsin, and then they were co-cultured with conventional CD4^+^CD25^−^T cells for 24 hrs in a ratio of 1:1, and treated with anti-CD3 (5μg/ml) and anti-CD28 (2μg/ml) antibody for polyclonal activation of T cells, respectively [14–16]. The proliferative activity, apoptotic rate as well as secretive ability (IFN-γ and IL-4) of CD4^+^CD25^−^ T cells, and the expression of Foxp-3/CTLA-4, as well as the secretive ability of TGF-β of every subtype of Tregs were determined.

*In vitro*, Nrp-1^high^CD4^+^CD25^+^Tregs was isolated from normal mice spleen, and cultured with different doses (including 1,10,100,1000 and 10000 μg/ml) of tuftsin in the stimulated of LPS(100ng/ml) for 24 hrs.

### CCK-8 measurement

The proliferative activity of CD4^+^CD25^−^ T cells was determined by CCK-8 according to protocols provided by the manufacturer. The absorbance was read in microplate reader (Spectra MR, Dynex, Richfield, MN) at OD450 nm.

### Flow cytometric analysis

CD4^+^CD25^+^Tregs were stained with FITC-conjugated anti-mouse-CTLA-4 for 30 minutes at 4°C in the dark. For determination of intranuclear Foxp3, CD4^+^CD25^+^Tregs were suspended in 1 ml fixation/permeabilization solution for 2 hours at 4°C in the dark. After washing cells with 1×permeabilization buffer twice, CD4^+^CD25^+^Tregs were stained with FITC-conjugated anti-mouse/rat-Foxp3 for 30 minutes at 4°C in the dark. After washing CD4^+^CD25^+^Tregs with PBS twice, cells were analyzed by flow cytometer (Becton-Dickinson) after the following procedures. 5×10^5^-1×10^6^ CD4^+^CD25^−^ T cells were washed in PBS twice, and suspended in 200 μl 1×binding buffer, followed by 10 μl FITC-conjugated annexin-V to stain for 30 minutes at 4°C or 15 minutes at 25°C in the dark. 300 μl 1×binding buffer and 5 μl PI were added to stain for 5 minutes at 25°C in the dark again, and they were subjected to flow cytometric analysis by flow cytometer.

### Immunofluorescence microscopy

CD4^+^CD25^+^Tregs were incubated with a rabbit anti-mouse Nrp-1 antibody (Abcam, Cambridge, MA) for 20 minutes at 4°C, washed and incubated with FITC-conjugated goat anti-rabbit IgG (Jackson, Southern Biotechnology Associations and Molecular Probes) for 30 minutes at 4°C. The average mean fluorescence (pixels/area) was analyzed using ImageJ software, and statistical significance of samples (n>3) was assessed using a one-way ANOVA.

### Methylation-sensitive RT- PCR

the demethylation level of Foxp3-TSDR was determined by methylation-sensitive RT- PCR, which has been recounted by R. Tatura, *et al* [13].

### ELISA measurement

The supernatants were collected for measurement of tuftsin IFN-γ, IL-4 and TGF-β levels by ELISA kits, strictly according to the protocols provided by manufacturer. 100 μl of ortho-phosphoric acid was added to terminate the color reaction. Plates were read in microplate reader at OD 450. The standard concentration curve for tuftsin IFN-γ, IL-4 and TGF-β were plotted from 0 to 1000 pg/ml. Examination of all samples was run in quintuplicates.

### Statistical analysis

Data were represented as mean ± standard deviation (SD), and analyzed by software of SPSS 17.0 with a one-way ANOVA. Unpaired Student's *t*-test was used to evaluate significant differences between groups. A *P*-value of 0.05 or 0.01 was considered statistically significant. Survival rate in septic mice was evaluated by Kaplan-Meier via the log-rank test.

## Abbreviations

Nrp-1: neuropilin -1
Tregs: Regulatory T cells
CLP: cecal ligation and puncture
Foxp-3: forkhead/winged helix transcription factor- 3
CTLA-4: cytotoxic T lymphocyte associated antigen-4
TGF-β: transforming growth factor -β
TSDR: Tregs specific demethylated region
DCs: dendritic cells
Th: helper T cell
TGF-βm+: membrane associated transforming growth factor-β
